# Norovirus: new developments and implications for travelers’ diarrhea

**DOI:** 10.1186/s40794-016-0017-x

**Published:** 2016-01-12

**Authors:** Mark P. Simons, Brian L. Pike, Christine E. Hulseberg, Michael G. Prouty, Brett E. Swierczewski

**Affiliations:** 1 U.S. Naval Medical Research Unit No.6 (NAMRU-6), Venezuela Ave, Block 36, Callao 2, Lima, Peru; 2 Naval Medical Research Center – Asia (NMRC-A), PSA Sembawang Deptford Rd, Building 7-4, Singapore, 759657 Singapore; 3 U.S. Army Medical Research Unit - Kenya, Kericho Field Station, PO Box 1357 Hospital Road, Kericho, 20220 Kenya; 4 U.S. Naval Medical Research Unit No. 2 (NAMRU-2), Phnom Penh, Blvd Kim Il Sung, Khan Toul Kork Phnom Penh, Cambodia; 5 Department of Enteric Diseases, U.S. Army Medical Directorate - Armed Forces Research Institute of Medical Sciences AFRIMS), 315/6 Rajvithi Rd, Bangkok, 10400 Thailand

**Keywords:** Norovirus, Travelers’ Diarrhea, Acute gastroenteritis

## Abstract

Noroviruses are the leading cause of acute gastroenteritis in the United States and are responsible for at least 50 % of acute gastroenteritis outbreaks occurring worldwide each year. In addition, noroviruses have caused outbreaks on cruise ships, in nursing homes and hospitals, and in deployed military personnel, but its role in the etiology of travelers’ diarrhea is not well defined. The aim of this review is to describe the role of noroviruses in travelers’ diarrhea in terms of epidemiology, current diagnostics, treatment and vaccine development efforts. Studies have shown prevalence rates of noroviruses in travelers’ diarrhea cases ranging from 10–65 %. It is likely that norovirus prevalence rates are highly underestimated in travelers’ diarrhea due to rapid onset, short duration of the illness, limited availability of laboratory facilities, and the fact that most clinical laboratories lack the diagnostic capability to detect noroviruses in stool. Further, additional studies are needed to accurately determine the true prevalence rates of norovirus as an etiologic agent of diarrhea among travelers to different regions around the world. With the rapid progress in the development of a norovirus vaccine, travelers could serve as an ideal population for future norovirus clinical trials.

## Background

Travelers’ Diarrhea (TD) is the most common illness reported in international travelers from industrialized nations to low-income, developing nations [1, 2]. TD can be caused by multiple etiologic agents to include enteric bacteria (diarrheagenic *Escherichia coli*, *Shigella spp.*, *Campylobacter spp.*), viruses (noroviruses, adenoviruses, astroviruses) and parasites (*Giardia lamblia*, *Entamoeba histolytica*, *Cryptosporidium parvum*) [3, 4]. However, noroviruses (NoVs) are the number one cause of epidemic gastroenteritis characterized by vomiting and diarrhea and are responsible for at least 50 % of all acute gastroenteritis outbreaks worldwide [5, 6]. Though NoV has been associated with several global epidemics, the prevalence of NoV-associated TD is not well defined and the number of studies aiming to assess NoV as a primary etiologic agent responsible for TD has been limited [7, 8]. There have been many excellent reviews recently published on the epidemiology and impact of NoVs [8, 9] and we refer the reader to these reviews for their completeness, whereas in this article we will focus on what is known about the role of NoV in travelers and how this information can be used for pre-trip travel medicine consultations, diagnosis of NoV in acute gastroenteritis (AGE) cases from returning travelers, and future goals of prevention through vaccines and other preventative interventions.

### Epidemiology of norovirus: implications for travelers

NoVs are a large family of evolutionarily divergent viruses named after the prototype Norwalk virus discovered in Norwalk, Ohio in 1968 [9] and have emerged since their discovery as one of the leading causes of AGE in all populations, including travelers. The viral capsid is composed of two proteins, VP1 and VP2. VP1, which self-assembles when expressed in various cell culture models, is the primary antigen utilized in serological diagnostic assays [5, 10]. VP1 is also important in binding to host cells via the interaction of the protruding (P) domain with histo-blood group antigens (HBGA) and the P domain also undergoes both antigenic drift and shift that allows both immune evasion and give NoVs their pandemic potential [11]. VP2 appears to bind to VP1, possibly stabilizing the capsid especially when exposed to harsh environments.

NoVs exhibit considerable genetic diversity and are categorized into seven known genotypes (GI-GVII), of which GI, GII, and GIV have been found to cause disease in humans [9, 12]. GII is the most common NoV found in human gastroenteritis cases, encompassing approximately 85 % of cases where the remaining 15 % of identified NoV infections are caused by GI with only sporadic cases due to GIV. NoVs are further categorized into genotypic subtypes based on the sequence of the viral capsid protein gene for VP1 [9, 12]. Of the GII viruses, the most common genotypic subtype is GII.4 which has been circulating worldwide since the 1990s. Continuing viral evolution has led to the emergence of novel pandemic strains every two to three years that have been responsible for many outbreaks and replaced endemic NoV variants [12]. Since its emergence in 2012, the GII.4 Sydney variant is the predominant GII.4 subtype in circulation [12].

It is estimated that NoVs account for 23 million cases of AGE each year in the United States alone and many more in developing regions where sanitation and hygiene practices are less robust. While the majority of cases result in only mild infections, approximately 500–700 cases in the U.S. per year are severe enough to lead to death [13]. In the U.S., individuals older ≥ 65 years old were most at risk for death from NoV infection while children < 5 years was the predominant age group for those individuals requiring medical attention [13]. People at risk for NoV infection include those living in close quarter facilities such as nursing homes and cruise ships, living conditions where food sanitation/hygiene practices are unsanitary and those individuals with an impaired immune system [9]. NoVs, which have a low infectious dose of an estimated 100 virions, are highly transmissible via the fecal-oral route [9, 14]. However, due to the low infectious dose and common symptoms of both vomiting and diarrhea, there have been suggestions that transmission can also occur via aerosolized vomitus or fecal matter [15]. Due to its ability to resist desiccation and disinfection, NoV is able to survive for long periods on surfaces allowing for transmission via fomites. These characteristics give NoV the propensity to cause secondary infections leading to its high potential to cause outbreaks of AGE in closed group settings. Many studies have examined NoV transmission in hospital settings, long-term care facilities, schools, and military camps, but less is known about the role of NoV in travelers. With the increasing rates of leisure travel especially to developing regions of the world, TD is a frequently encountered concern for travelers. Additionally, politicians, diplomats, and military personnel frequently travel to regions of high-risk for TD and prevention of illness is important for mission accomplishment. Given the possible association between NoV acute infection and the subsequent development of post-infectious diarrhea sequelae such as irritable bowel syndrome (IBS), avoiding infection may be important for long term health [1].

There are several studies that have shed light on the role of NoV in TD among travelers. In a study of 3 different cohorts of U.S. European travelers (84.2 % male, mean age 28.8 years) to Guatemala, Mexico or India, NoV during various periods between 2002–2007, in combination was detected in 10.2 % of moderate to severe diarrhea cases (at least 3 or more unformed stools and other symptoms) and was the second most common pathogen detected following diarrheagenic *E. coli* (ETEC and EAEC) [16]. Prevalence of NoV in this study varied from 3–17 % among the different regions (3–12 % from Mexico, 11.9 % from India, and 17 % from Guatemala) with many travelers found to have co-infections with bacterial and parasitic pathogens and 22.4 % infected with 3 or more pathogens [16] In another study of European travelers (52.9 % male, mean age 37 years) returning to Germany from over 50 worldwide locations, NoV was found in 15.7 % (9/57), persons with diarrhea and 1/47 asymptomatic persons [17]. Additionally, backpacking trips had the highest association with NoV infection (OR =4.9), followed by business trips (OR = 1.4), and packaged vacations with the lowest association (OR = 0.3) [17]. The study by Apelt, et al. [17] demonstrated that of the 9 NoV cases detected, onset of symptoms ranged from 10 days prior to return to up to 4 days after returning, suggesting that testing patients as much as a week after the onset of diarrhea, especially if experiencing continuing symptoms, is a worthwhile strategy. Other studies of travelers to various regions around the world have shown NoV prevalence ranging from 15–65 % with varying genotypes detected [5, 17–22]. Additionally, NoV has been a well-documented cause of outbreaks on cruise ships and transmission has been observed during air travel [9, 15, 23]. TD has been traditionally thought to be caused by bacterial pathogens [24], but with the association of NoV among many different food types and water sources [24, 25]; the risk of exposure to NoV in travelers is high and should be considered in all cases of TD. Finally, since NoV has been shown to be shed in stools for as much as 56 days after symptoms have resolved, enhanced awareness of sanitation and hygiene among returning travelers who experienced TD is necessary to avoid secondary cases among contacts.

### Symptoms and clinical outcomes

The clinical course of NoV infection has been described in detail in many reviews [1, 9], but summarized here as it relates to disease in travelers who are usually relatively healthy and not commonly among the extreme age groups (young and old) that may be more vulnerable to infection and complications. In healthy adults, infection by NoV is typically acute and self-limiting, but causes significant morbidity that arises from a combination of symptoms including the classic symptoms of vomiting and diarrhea as well as accompanying symptoms such as abdominal cramping, fever, and malaise. Symptoms typically emerge between several hours and two days post-infection and resolve within 72 h of presentation [26].

Of 23 confirmed NoV-attributable cases reported in U.S. students who traveled to Mexico between 2007 and 2008, the recorded symptoms included abdominal cramping, vomiting, and severe levels of nausea and fecal urgency [16]. Similarly, abdominal cramping (95 %), nausea (74 %), and fecal urgency (63 %) were experienced in a majority of the NoV-confirmed cases in a separate study on U.S. and European travelers returning from Guatemala, India, and Mexico [26]. Descriptions of NoV-attributable gastroenteritis among deployed military personnel have also reported similar symptoms. A 2002 NoV outbreak of >1,300 British military personnel in Iraq cited high levels of diarrhea (89 %), abdominal pain (85 %), nausea (80 %), and vomiting (56 %), with an average hospitalization of 1.6 days [27]. Another NoV outbreak among U.S. military personnel in Turkey involved multiple genotypes and showed a similar pattern with 79 % of individuals having diarrhea, 77 % abdominal cramping, 63 % nausea, and 46 % vomiting [28].

While infection typically results in mild to moderate symptoms of short duration, a more prolonged and severe clinical course has been described in infants, the elderly, and among immunocompromised individuals [26, 29, 30]. Beyond the acute illness itself, NoV has also been associated with an increased risk of post-infectious sequelae. Diagnostic surveys returned after a waterborne outbreak in Italy in 2009 indicated that 40/348 diarrhea cases (12 %) went on to develop IBS. At 12 months post-infection, the odds of developing IBS was >10-fold higher in the exposed group as compared to unexposed controls [31]. At the same time, a large retrospective cohort study spanning eight years that involved 1,718 cases of NoV infection estimated an increased risk of dyspepsia, constipation, and gastroesophageal reflux disease (GERD) following infection [32]. Compared to unexposed controls, increased relative risk (RR) was noted for dyspepsia (RR = 1.44; 95 % CI: 0.84–2.47), constipation (RR = 1.32; 95 % CI: 0.96–1.81), and GERD (RR = 1.39; 95 % CI: 1.07–1.81), in the exposed outbreak population. Interestingly, in contrast to the aforementioned IBS studies, this same study found a decreased risk for IBS among infected individuals rather than an increased risk for development of IBS (RR = 0.68; 95 % CI: 0.30–1.51) [32]. Other reports have also hinted at possible connections between infection and the development of reactive arthritis [33] and exacerbations of inflammatory bowel diseases (IBD), although this has been debated in other reports [34, 35].

Epidemiologic evidence in favor of an association between NoV infection and the development of post-infectious conditions has been accumulating slowly over the past decade, and the supporting data are congruent with studies linking bacterial causes of gastrointestinal illness to chronic sequelae [36, 37]. However, further studies are needed to establish a causal link. Elucidating the relationship between NoV and the development of any associated sequelae will require well-designed prospective studies that examine the strength of the association, any temporal and dose–response relationships that may exist, and the consistency of the effect of NoV infection on the postulated outcome. At the same time, these studies should take into account host factors that may determine the relative risks of different populations, including travel populations, and any potential differential influence that disparate genogroups and strains of NoV may have on a particular outcome.

### Diagnostics and testing strategies

Understanding the biology, transmission, and epidemiology of NoV has been limited by a lack of reliable and cost-effective diagnostics. Much of the struggle to understand the biology and burden of NoV is due to the inability to culture the virus [12] with some studies resorting to the use of surrogate viruses (feline or murine calciviruses) to assess virus viability after treatment with disinfectants. Lack of culture capability limits investigation into the biology and pathogenesis of NoV and also prevents large scale amplification to create pure viral stocks used for internal controls in laboratory tests, thus NoV must be stored in frozen stools. A recent breakthrough by Jones et al. allowing for culture of NoV in B cell lines with the addition of HBGA-bearing bacteria may have significant impact for future NoV diagnostics [38, 39], but is not available in most clinical laboratories. Thus other methods for detection are commonly used including enzyme-linked immunosorbant assays (ELISA) for NoV antigen detection or reverse-transcription polymerase chain reaction (RT-PCR) to detect specific NoV genes. For these assays, the preferred specimen is fresh or frozen stool. Vomitus can also be tested if collected and processed immediately. Testing of food and environmental surfaces is also possible, but low numbers of viral particles and potential inhibitors may affect the sensitivity of detection.

Recently, there have been excellent reviews of the performance of both commercial and laboratory developed ELISAs and RT-PCR assays for detection of NoV and we refer the reader to these sources for a complete description of the performance of available assays [9, 12]. As discussed in these reviews, the performance of ELISAs a for antigen detection is limited by sensitivity based on viral load and ability to detect different genotypic subtypes. Therefore a recommendation is to test multiple stools to increase sensitivity, a strategy often employed during outbreak situations where a few positive samples are sufficient for source attribution [9, 12].

Because of the poor sensitivity of NoV antigen detection by ELISA, molecular detection of NoV has become the preferred method. Most RT-PCR assays use primers that target the conserved region between ORFs 1 and 2 with good sensitivity and specificity [9, 12]. Although RT-PCR assays require technical expertise of laboratory staff and are expensive, they are becoming increasingly cost-effective as more commercialized assays become available. New multiplex molecular assays targeting the common causes of infectious enteric diseases including NoV have been developed including three FDA-approved multiplex assays (Biofire FilmArray Gastrointestinal Panel, Luminex Gastrointestinal Pathogen Panel, and Nanosphere Verigene Enteric Pathogens Test) [9, 12]. The advantage of the multiplex assays is detection of multiple pathogens (7–23 depending on the assay chosen) which can improve overall detection of causative etiology, especially with viruses [40]. Adding molecular detection for NoV and other enteric viruses is beneficial considering between 40–60 % of samples submitted to laboratories do not yield an identified pathogen for a variety of reasons including sample collection procedures, transport time, and low pathogen levels [41]. As multiplex molecular testing becomes more commonplace in diagnostic laboratories, a more complete understanding of the complex etiology of TD will be determined. Many studies have reported co-infections when using multiplex methods with NoV often found with other enteropathogens raising questions about how these complex interactions affect disease duration, severity, and post-infectious outcomes.

With improvements in the costs, ease of use, and availability of molecular testing in diagnostic laboratories, clinicians should be encouraged to submit samples for testing from returning travelers with current symptoms of AGE and also those reporting disease in the previous week. NoV should be considered in the differential diagnosis of all returning travelers and tests for NoV should be requested in addition to standard stool culture. This is important not only for treatment of the individual patient, but in cases which are found positive for NoV, the results would allow guidance on continued focus on hygiene and proper disinfection to avoid spread among cohabitants of infected travelers since NoV can be shed in stools for a long duration after symptoms resolve [9]. Similarly, NoV should be the primary consideration of all persons with AGE reporting contact with a recent returning traveler.

### Treatment and prevention

Between 12 % and 46 % of travelers are forced to alter their travel plans because of acute diarrheal infections [1]. However, efforts to develop targeted therapeutics for NoV infections have long been stagnated by the lack of appropriate small animal models and the infeasibility of propagating NoV in cell culture [42]. While NoV treatment options have largely centered on supportive care [9, 26], there is some impetus for broadening treatment options even among the healthy adult population. In the sense that they have critical or itinerant activities that necessitate rapid relief of the discomfort of acute diarrhea, some travelers may be grouped alongside emergency workers, military service members, and other priority groups. The particularly high transmissibility of NoV also increases pressure to identify drugs that can mitigate its outbreak potential. Otherwise healthy travelers suspected of suffering from NoV-caused diarrhea are generally given oral rehydration therapies and bismuth subsalicylate (Pepto-Bismol) [9, 16]. Anti-motility agents such as loperamide and diphenoxylate plus atropine may also be included in the limited arsenal of non-antibiotic treatment options for viral gastroenteritis, although caution must be exercised in instances where co-infections with enteroinvasive dysenteric pathogens are suspected [43]. However, in several studies involving both children and adults, subjects with gastroenteritis who received loperamide versus placebo reported no significant difference in the incidence of adverse effects in either treatment arm [44].

In instances where oral rehydration efforts have failed and the subject runs the risk of dehydration, antiemetic drugs (commonly used to prevent nausea in patients undergoing chemotherapy) have also been evaluated as both a treatment of AGE and as a transmission control measure in pediatric populations [44–46]. In one such treatment trial involving ondansetron, less than half of the children receiving oral ondansetron required intravenous hydration compared to the placebo control group [47]. Measures to control vomiting may also be important in controlling person-to-person transmission of NoV among travelers, particularly in the classic setting of a cruise ship outbreak [48, 49].

Among the few targeted, virus-specific approaches against NoV infection under investigation is nitazoxanide, a compound licensed in the US for the treatment of cryptosporidiosis but with broad-spectrum activity against a number of protozoal and helminthic parasites and bacteria [50]. A phase II treatment trial involving children with severe rotavirus AGE found the duration of diarrheal illness in children reduced by over two-fold relative to a placebo-controlled cohort [51]. A clinical trial of nitazoxanide efficacy in thirteen NoV-confirmed cases of AGE showed a significantly shortened time to resolution of symptoms (1.5 days versus 2.5 days) in subjects who received 500 mg of nitazoxanide for three days compared to a placebo group [52]. Having been licensed as an anti-parasitic compound for nearly 15 years, an estimated 75 million Americans have been exposed to nitazoxanide without any serious adverse effects reported [53, 54] and, in part due to its broad-spectrum applications, the drug is now being considered as an essential medicine for neglected tropical diseases [55]. Further data from clinical trials involving nitazoxanide including a comparison to loperamide for efficacy may provide clinicians travelers with a future treatment option for NoV infections acquired during travel.

Due to the highly infectious nature of NoV and rapid impact on functionality, all travelers should be counseled during their pre-travel consultations on diligence of hand hygiene prior to eating, use of bottled water at all times including when brushing teeth, and reducing close contact with persons experiencing symptoms of AGE. Additional discussions should include an evaluation of the type of travel (adventure/backpacking, business, etc.) and elaboration of the level of risk associated with the traveler’s plans and potential prevention strategies. Furthermore, advice to the traveler, as with any cause of diarrhea proper hydration that includes electrolytes of equivalent volume to that lost through diarrhea and vomiting is the best treatment during the duration of symptoms. Travelers should also be encouraged on sanitation and hygiene discipline when sick to avoid spreading among their group and to other travelers.

### Vaccine

The development of a safe efficacious vaccine to protect both travelers and non-travelers against NoV is considered one of the most feasible and economical approaches to limiting the potential impact of NoV infections. Currently there are no approved vaccines directed against NoV and development of a vaccine has been hampered by several challenges highlighted below. With the current inability to culture NoV and develop attenuated vaccine strains in the laboratory setting, vaccine efforts have instead focused on the development of other platforms. Jiang, et al. reported in 1992 that the VP1 protein of NoV, when expressed in cell culture, is able to self-assemble into virus-like particles (VLPs) [56]. With structural and antigenic properties similar to native virus but in a noninfectious, non-replicating form, these VLPs were identified as potential vaccine candidates and many studies have been conducted over the last 10 years showing good serum responses and initial efficacy, providing the foundation for recent promising studies.

In an effort to develop a VLP-based vaccine with broad coverage against circulating NoVs of both the GI.1 and GII.4 genotypes, Treanor, et al. conducted testing on a bivalent GI.1- GII.4 consensus VLP vaccine delivered by the intramuscular route. Subjects were given 5, 15, 50 or 150 μg of each VLP vaccine 28 days apart. Initial safety and immunogenicity studies indicated the vaccine was well tolerated and a robust immune response was stimulated with 100 % of subjects responding to GI.4 VLP vaccine at any dose level and 56–88 % responding in a dose dependent manner to GII.4 consensus VLP [57]. The vaccine stimulated a strong serum antibody and antibody-secreting cell (ASC) response following one dose. However, the early robust ASC response is indicative of a recall response by memory B cells as opposed to a naïve immune response [58]. This finding is not surprising given the high likelihood that the human volunteers had previously been exposed to NoV through natural infection. While the vaccine stimulated a robust immune response in what are presumably previously exposed subjects, how the vaccine would perform in naïve individuals remains to be assessed.

As a follow on to the previous trial, a second study by Bernstein*,* et al. utilized a bivalent vaccine composed of 50 μg of GI.1 VLPs and 50 μg of consensus GII.4 VLPs with monophosphoryl lipid A (MPL) and alum adjuvants [59]. Two intramuscular injections were given 28 days apart. Patient immune responses were monitored and subjects were challenged with GII.4 NoV at a minimum of 28 days following the second vaccination. No severe adverse events were recorded as a result of vaccination. Twenty eight days following the first vaccination subjects exhibited a 62.2-fold increase in ELISA geometric mean titers (GMTs) to GI.1 and a 10.8-fold rise in ELISA GMT to GII.4. However, no significant increase in antibody titers was detected following the second dose. One hundred percent of vaccinated subjects demonstrated a seroresponse against GI.1, whereas the seroresponse to GII.4 peaked at 89.8 % as compared to only 2.1 % of subjects given placebo injections demonstrated a seroresponse [59]. Despite the promising immunological response, the vaccine did not significantly reduce the incidence of protocol-defined illness of gastroenteritis, possibly due to an overly strict definition combined with under self reporting of symptoms. However, vaccinated subjects did demonstrate a reduction in self-reported severe, moderate or greater, and mild or greater severity of vomiting and/or diarrhea [59]. Taken together these data may indicate that a NoV vaccine may provide better protection against severe manifestations of the disease rather than moderate or milder infections, not unlike the results seen with the rotavirus vaccine.

The economic feasibility for the development of a NoV vaccine is comparable to many current vaccines in that long lasting protection with high efficacy is required to offset vaccination costs. When vaccine cost effectiveness was modeled based on the estimated US NoV burden, it was determined that a vaccine that provided only 12 months of protection would not result in cost savings; however, if the vaccine protected for at least 48 months (≥50 % efficacy/≤$25 or ≥75 %/≤$50), the overall healthcare costs savings would be $100 million to $2.1 billion a year [60]. In the U.S. military, itself a unique travel population, NoV has been a major cause of duty days lost due to AGE in deployed operations. Using the parameters outlined in their model, Tallant et al. estimated that a NoV vaccine would cost $1344 per duty day lost averted (e.g. subject able to perform duties versus being ill). However, the authors state that the monthly incidence and pathogen prevalence for NoV are most likely under-reported and when the model was adjusted to include illness with vomiting, the cost per duty day lost averted dropped to $572, which is fiscally favorable when compared to the U.S. military operational costs estimate of $935 per day per deployed troop. Additionally, the authors indicate that a NoV vaccine would enhance troop safety and well-being as distractions due to gastrointestinal illness in deployed settings would be minimized [61]. Clearly, a long lasting, efficacious vaccine directed against NoV would be able to provide significant domestic healthcare savings and also provide a beneficial cost benefit ratio for operations in deployed conditions where gastrointestinal illness can jeopardize the mission. An efficacious vaccine would benefit travelers as the likelihood of significant NoV outbreaks occurring particularly on cruise ships would be greatly reduced and a NoV vaccine could eventually become part of a traveler’s clinician recommendations for travelers.

## Conclusions

NoV will continue to remain a pathogen of critical importance as a major cause of AGE worldwide. With NoV incidence in travelers being likely underestimated due to challenges in NoV diagnostics, improvement in molecular methods for identification NoV will enable future TD studies to adequately measure NoV as a burden of AGE and also allow for a better clinical diagnosis of NoV in a hospital setting. Future studies aimed at further characterizing the epidemiology of NoV in Western travelers should be undertaken as travelers could prove to be a viable population for future NoV vaccine trials.
